# Supplementary material for: Higher native Peruvian genetic ancestry proportion is associated with tuberculosis progression risk
Source: Cell Genom. 2022 Jun 27;2(7):100151. doi: 10.1016/j.xgen.2022.100151 (PMC9306274; doi:10.1016/j.xgen.2022.100151)
Supplement: Document S1. Methods S1–S4, Figures S1–S8, and Tables S1–S13 [file mmc1.pdf]

Cell Genomics, Volume *2*

Supplemental information

Higher native Peruvian genetic ancestry proportion is associated with tuberculosis progression risk

Samira Asgari, Yang Luo, Chuan-Chin Huang, Zibiao Zhang, Roger Calderon, Judith Jimenez, Rosa Yataco, Carmen Contreras, Jerome T. Galea, Leonid Lecca, David Jones, D. Branch Moody, Megan B. Murray, and Soumya Raychaudhuri

Table of contents

Methods S1: Genotyping array design, Related to STAR Methods. 2
Methods S2: Merging genotyping data from our Peruvian cohort with data from native American and Siberian individuals, Related to Figure 2A and STAR Methods. 2
Methods S3: Merging genotyping data from our Peruvian cohort with data from native American and Siberian individuals and the 1000 Genomes Project, Related to Figure 2A and STAR Methods. 3
Methods S4: Merging genotyping data from our Peruvian cohort with data from the 1000 Genomes Project, Related to Figure 2A and STAR Methods. 4
Supplementary Figures 5
Figure S1: Flowchart of the study design, Related to STAR Methods. 5
Figure S2: Geographical distribution of TB cases and TST positive HHCs, Related to STAR Methods. 6
Figure S3: Principal Component Analysis (PCA), Related to STAR Methods. 7
Figure S4: Global ancestry inference results using different reference panels, Related to Figure 2A. 9
Figure S5: ADMIXTURE analysis results using K = 4-7 clusters, Related to Figure 2A. 10
Figure S6: PC-relate pairwise kinship coefficients, Related to STAR Methods. 11
Figure S7: Proportion of native Peruvian genetic ancestry in males and females, Related to Figure 2B. 12
Figure S8: Admixture mapping following local ancestry inference using PCAdmix, Related to STAR Methods. 13
Supplementary Tables 15
Table S1: Global ancestry inference results using Global ancestry inference results using different reference panels, Related to Figure 2A. 15
Table S2: Ancestral proportions when using a larger number of ancestral clusters (K = 4-7) in the ADMIXTURE analysis, Related to Figure 2A. 16
Table S3: Self-reported race and ethnicity in our cohort among individuals with > 0.9 estimated native Peruvian genetic ancestry (N = 985), Related to Figure 3. 17
Table S4: Self-reported race and ethnicity in our cohort among individuals with < 0.5 estimated native Peruvian genetic ancestry (N = 140), Related to Figure 3. 18
Table S5: Self-reported race in native peruvian ancestry tertiles for cases and controls, Related to Figure 3. 19
Table S6: Association between self-reported race and TB progression, Related to Figure 3. 21
Table S7: Accounting for self-reported race, Related to Table 3. 22
Table S8: Association of genetic ancestry and TB progression risk among unrelated individuals (N= 1929 TB cases and 1066 HHCs), Related to Table 3. 23
Table S9: Sex-stratified analysis, Related to Table 3. 24
Table S10: Testing the association between native Peruvian ancestry and TB progression risk using larger numbers of ancestral clusters (K = 5-7), Related to Table 3. 25
Table S11: Association of native Peruvian ancestry with TB progression risk after correction for potential confounders, Related to Table 3. 26
Table S12: Sensitivity analysis using microbiologically confirmed TB cases and their HHCs that were TST positive at baseline, Related to Table 3. 28
Table S13: Variants at the 5p23.2 locus that were nominally associated with TB progression risk in our previously published GWAS, Related to STAR Methods. 29

Methods S1: Genotyping array design, Related to STAR Methods.

We used exome sequencing data from 116 Peruvian TB cases from the same population as our study population in the design of our genotyping array (LIMAArray) in order to optimally capture Peru's genetic variation, and particularly rare and protein coding variations. We combined the content from the Affymetrix Axiom® myDesign custom genotyping array with our exome sequencing data, and whole-genome sequencing data from 75 Peruvians from the 1000 Genomes project. We then selected 302,400 markers including all coding variants, variants that were more frequent in Peruvians compared to other populations in the 1000 Genomes project, and variants in the proximity of known TB genes. Additionally, we included 8,100 markers from GWAS catalogue based on known or suggestive associations with autoimmune diseases, TB phenotypic variation, as well as markers from the HLA region. We also included 4,500 known ancestry informative markers. Finally, we included 397,200 markers that provide good genome-wide coverage for imputation in Peruvian populations in the common (>5%), low frequency (1–5%) and rare (0.5–1%) MAF ranges, including many regulatory variants. All together this resulted in an array with 712,200 markers. For more details about LIMAArray and its performance see reference.

Methods S2: Merging genotyping data from our Peruvian cohort with data from native American and Siberian individuals, Related to Figure 2A and STAR Methods.

1- We used convertf to convert the original files including 364,470 variants to plink format.

2- We used liftOverPlink (https://github.com/sritchie73/liftOverPlink) to lift data from hg18 to GRCh37; 364,396 variants were lifted successfully.

3- We used plink v1.90b to update variant IDs to chromosome:position:allele1:allele2

4- We started from 677,232 bi-allelic variants and used plink v1.90b to update variant IDs to chromosome:position:allele1:allele2

5- We used plink --bmerge function to merge the Reich data (see Reich et al 2012 in the main references) and TBRU data

6- 9,907 variants failed merge due to having multiple alleles

7- We removed the failed variants and redid the merging

8- The merged file includes 994,444 variants

9- We applied the following QC measures on the merged file using plink v1.90b genotyping missingness < 5% (--geno 0.05) minor allele frequency > 1% (--maf 0.01) and Hardy-Weinberg equilibrium p-value > 10^{-5} (--hwe 10e-5) in controls applying these filters led to 34,958 post-QC variants that were present in both datasets

10- We then pruned the data for linkage disequilibrium (LD) by removing the markers with $r^2 > 0.1$ with any other marker within a sliding window of 50 markers per window and an offset of 10 using PLINK. The final merged dataset included 23,169 variants.

Methods S3: Merging genotyping data from our Peruvian cohort with data from native American and Siberian individuals and the 1000 Genomes Project, Related to Figure 2A and STAR Methods.

1- We started from 81,083,551 bi-allelic variants from the 1000 Genomes Project phase 3 and used plink v1.90b to update variant IDs to chromosome:position:allele1:allele2

2- We then merged the 1000 Genomes Project variants with the 34,958 post-QC from the above analysis resulted in 81,117,189 variants

3- We applied the following QC measures on the merged file using plink v1.90b genotyping missingness < 5% (--geno 0.05) minor allele frequency > 1% (--maf 0.01) and Hardy-Weinberg equilibrium p-value > 10^{-5} (--hwe 10e-5) in controls applying these filters led to 34,936 post-QC variants that were present in both datasets

4- We then pruned the data for linkage disequilibrium (LD) by removing the markers with r^2 > 0.1 with any other marker within a sliding window of 50 markers per window and an offset of 10 using PLINK. The final merged dataset included 22,198 variants.

Methods S4: Merging genotyping data from our Peruvian cohort with data from the 1000 Genomes Project, Related to Figure 2A and STAR Methods.

1- We started from 677,232 bi-allelic variants from our Peruvian cohort and 81,083,551 bi-allelic variants from the 1000 Genomes Project phase 3 with variant IDs in both files set to chromosome:position:allele1:allele2

2- We selected 521,779 the variants that were present in both datasets from each dataset

3- we merged the two datasets using the shared variants

4- We applied the following QC measures on the merged file using plink v1.90b genotyping missingness < 5% (--geno 0.05) minor allele frequency > 1% (--maf 0.01) and Hardy-Weinberg equilibrium p-value > 10^{-5} (--hwe 10e-5) in controls applying these filters led to 272,531 post-QC variants that were present in both datasets

5- We then pruned the data for linkage disequilibrium (LD) by removing the markers with r^2 > 0.1 with any other marker within a sliding window of 50 markers per window and an offset of 10 using PLINK. The final merged dataset included 120,630 variants.

Supplementary Figures

Figure S1: Flowchart of the study design, Related to STAR Methods.

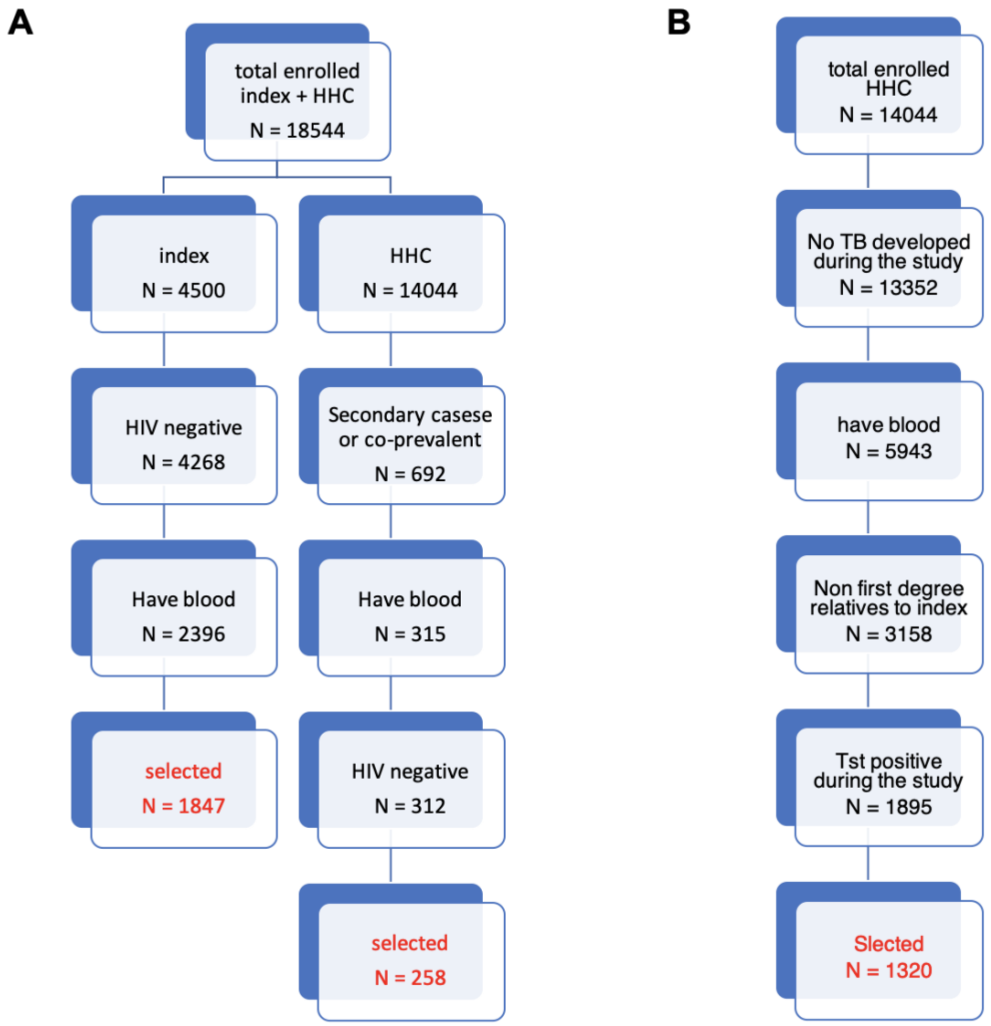

The cohort presented in the current study is a subset of a much larger epidemiological cohort (see Becerra et al 2019 and Odone et al 2016 in the main references). The flowcharts describe **A)** the selection of TB cases (N=2105) and **B)** TST positive HHCs (N=1320) for the current study from the larger cohort. TST: tuberculin skin test.

Figure S2: Geographical distribution of TB cases and TST positive HHCs, Related to STAR Methods.

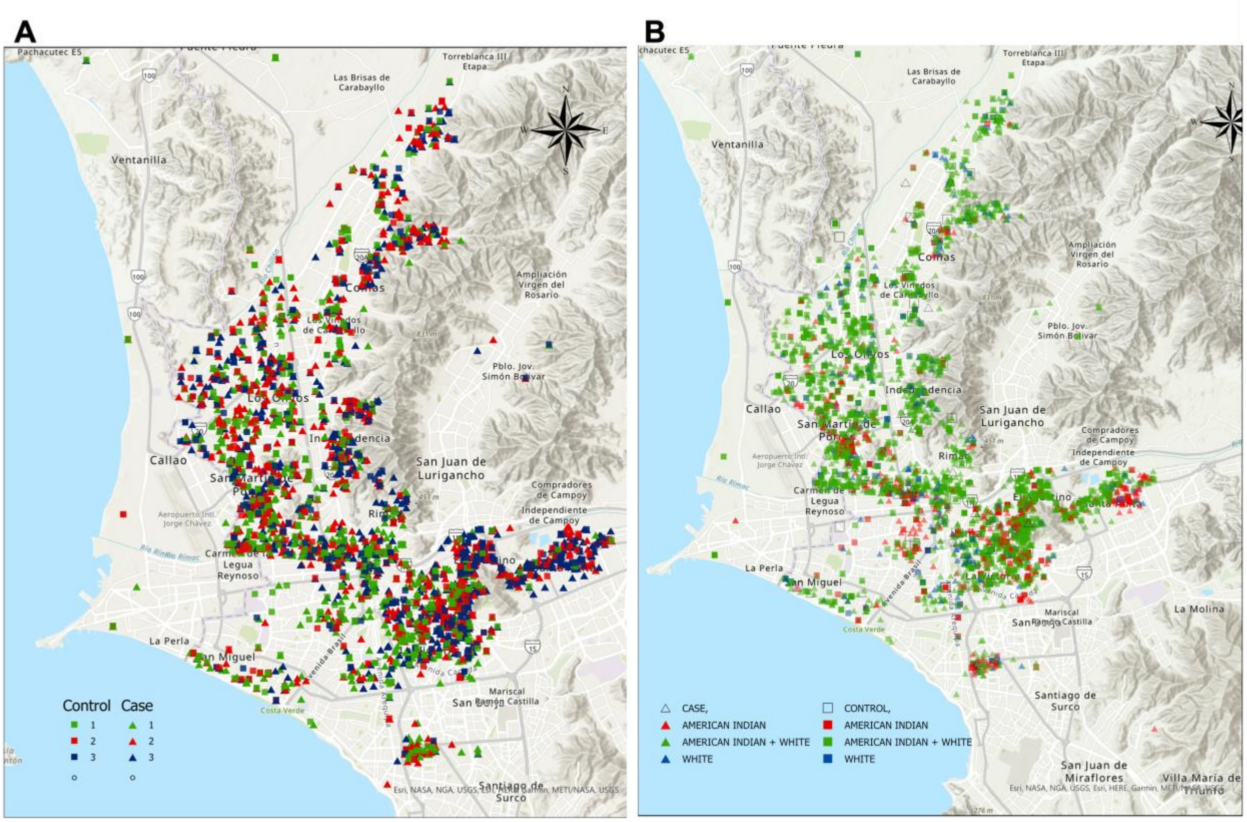

A) Geographical location of TB cases and their TST negative HHC (controls) based on tertiles of native Peruvian genetic ancestry. The proportion of native Peruvian genetic ancestry in the first, second, and third tertiles are 0.64 (sd=0.13), 0.84 (0.03), and 0.94 (0.03) respectively. **B)** Geographical location of TB cases and their TST negative HHC (controls) based on the most common categories of self-reported race: “American Indian + White”, “American Indian”, “White”.

Figure S3: Principal Component Analysis (PCA), Related to STAR Methods.

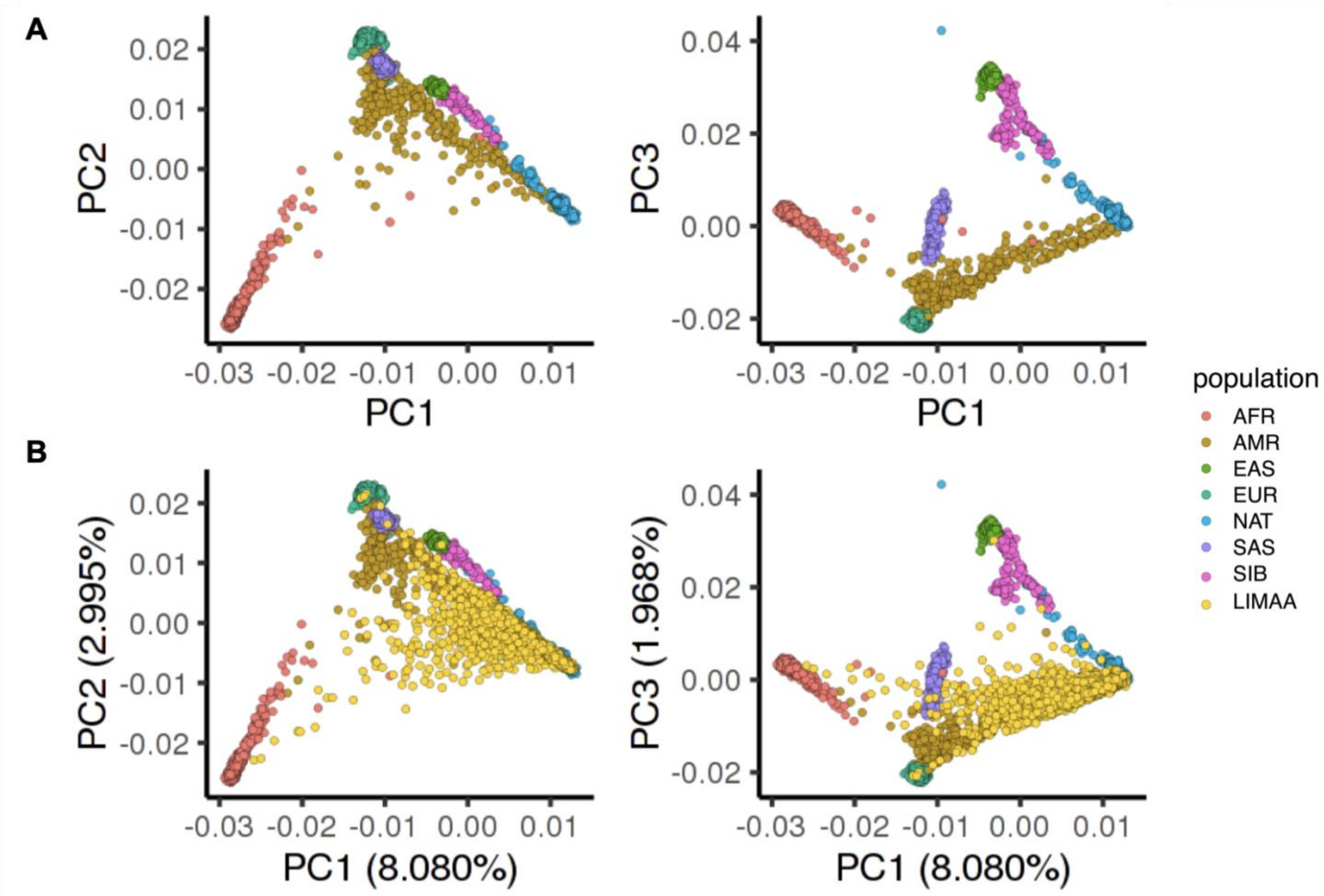

PCA analysis of genotyping data from Peruvians included in this study (LIMAA, N = 3425) merged with the data from populations from the 1000 Genomes Project phase 3 (N = 2054) as well as the data from Siberian and native American populations (N = 738) as reference panel (number of variants = 34936). **A)** PCA is shown without Peruvians from the current study to make visualization of reference populations easier. **B)** Peruvians from the current study are shown in yellow. Each individual is represented as a dot. Populations are colored based on their continental origin for or based on their assignment to native American or Siberian tribes. AFR: African ancestry includes: Yoruba in Ibadan, Nigeria, Luhya in Webuye, Kenya, Gambian in Western Divisions in the Gambia, Mende in Sierra Leone, Esan in Nigeria, Americans of African Ancestry

in SW USA; EUR: European ancestry, includes: Central European, Utah Residents (CEPH) with Northern and Western European Ancestry, Toscani in Italy, Finnish in Finland, British in England and Scotland, Iberian Population in Spain; EAS: East Asian, includes: Han Chinese in Beijing, China, Japanese in Tokyo, Japan, Southern Han Chinese, Chinese Dai in Xishuangbanna, China, Kinh in Ho Chi Minh City, Vietnam; SAS: South Asian, includes: Gujarati Indian from Houston, Texas, Punjabi from Lahore, Pakistan, Bengali from Bangladesh, Sri Lankan Tamil from the UK, Indian Telugu from the UK; PUR: Puerto Ricans from Puerto Rico; CLM: Colombian from Medellin, Colombia; MXL: Mexicans from Los Angeles, California; PEL: Peruvians from Lima, Peru. Altic: Altaic language family, includes: Yakut, Buryat, Evenki, Tuvinians, Altaian, Mongolian, Dolgan. North Amerind: Northern Amerindian language family, includes: Maya, Mixe, Kaqchikel, Algonquin, Ojibwa, and Cree. Central Amerind: Central Amerindian language family, includes Pima, Chorotega, Tepehuano, Zapotec, Mixtec, and Yaqui. Andean: Andean language family, includes Quechua, Aymara, Inga, Chilote, Diaguita, Chono, Hulliche, and Yaghan. For a full list of all populations in all language groups see Reich et al 2012 in the main references.

Figure S4: Global ancestry inference results using different reference panels, Related to Figure 2A.

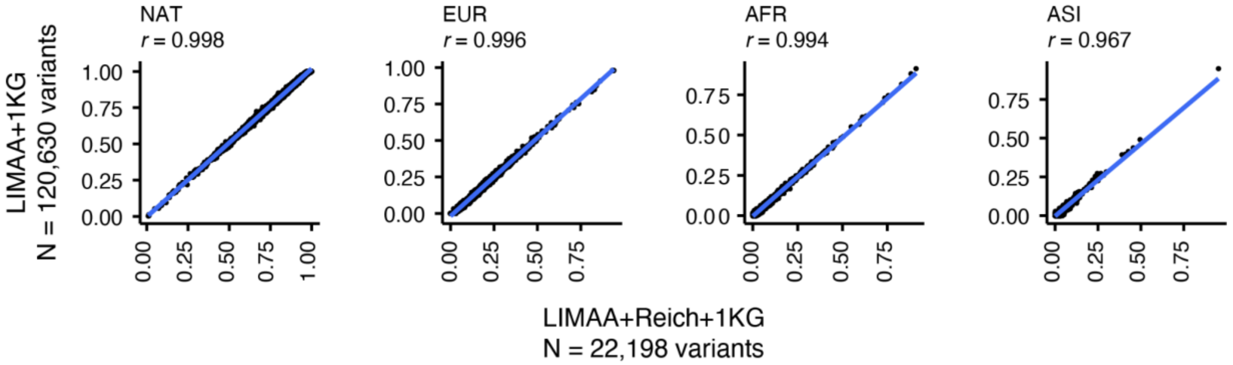

Correlation between global ancestry estimates using ADMIXTURE (Alexander et al., 2009) (K = 4) analysis with data from Peruvians included in this study (LIMAA, N = 3425) merged with the data from populations from the 1000 Genomes Project phase 3 (1KG, N = 2054) or the data from populations from the 1000 Genomes Project phase 3 plus Siberian and Native American populations (N = 738) as the reference panel. Each dot represents one individual. r= Pearson correlation coefficient. NAT: native Peruvian genetic ancestry, EUR: European genetic ancestry, AFR: West African genetic ancestry, ASI: East Asian genetic ancestry.

Figure S5: ADMIXTURE analysis results using K = 4-7 clusters, Related to Figure 2A.

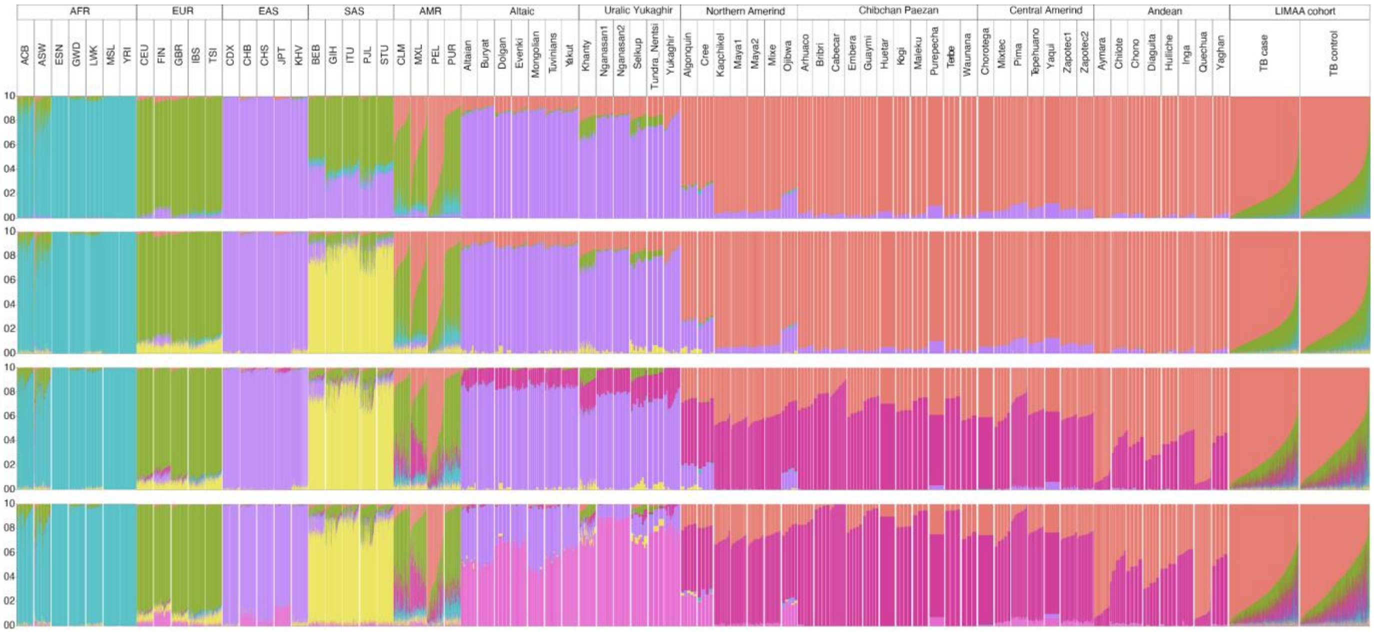

Admixture analysis for K= 4-7 clusters. Y-axis: genomic proportion. Column labels: population name and supergroup. The last two columns represent TB cases and controls from this study (N = 2105 and 1320 respectively). EUR: European, AFR: African, SAS: South Asian, EAS: East Asian, AMR: Central/South American. Due to space limitation, not all populations from are shown.

Figure S6: PC-relate pairwise kinship coefficients, Related to STAR Methods.

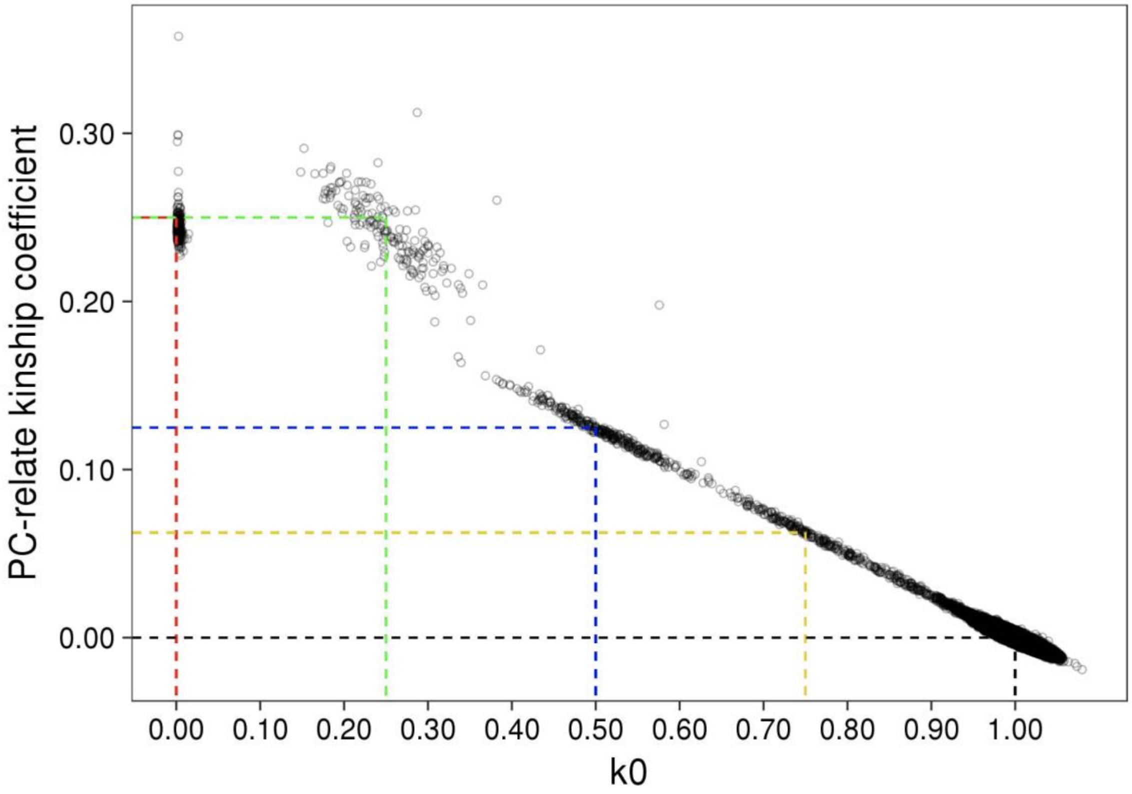

Of the total 551 related pairs, 36 pairs were parent-child (red dashed line), 72 were sib pairs (green dashed line), and 453 pairs were second-degree relatives (blue dashed line). The rest were more distant relatives (yellow dashed line) or unrelated (black dashed line). Y: PC-relate pairwise kinship coefficients. X: IBD sharing probability that the individuals share 0 copies of the alleles.

Figure S7: Proportion of native Peruvian genetic ancestry in males and females, Related to Figure 2B.

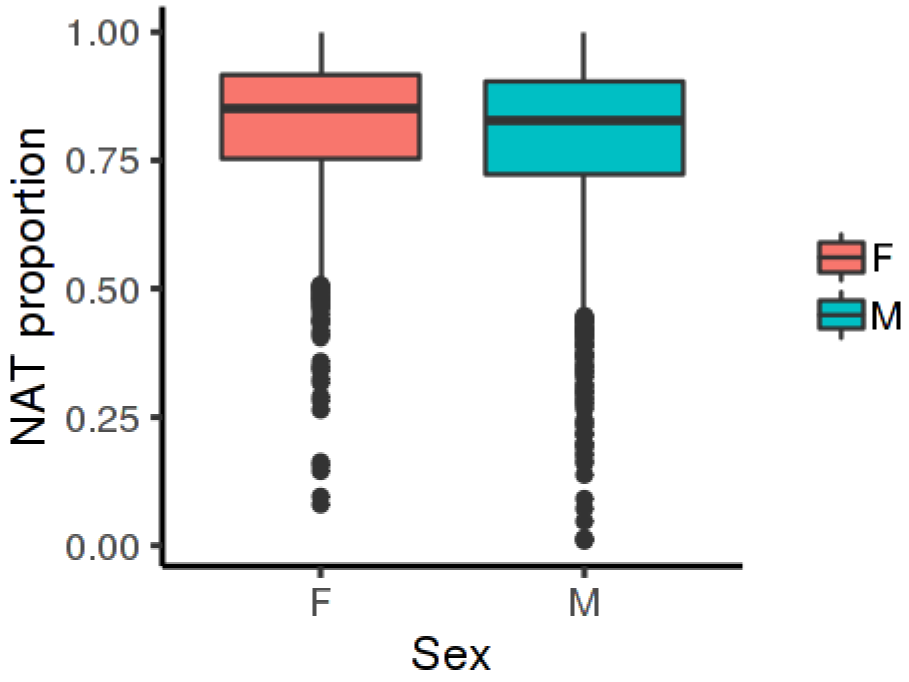

Females have slightly higher native Peruvian genetic ancestry that males (0.82 vs 0.79, t=test two-sided $p=4.5x10^{-10}$).

Figure S8: Admixture mapping following local ancestry inference using PCAdmix, Related to STAR Methods.

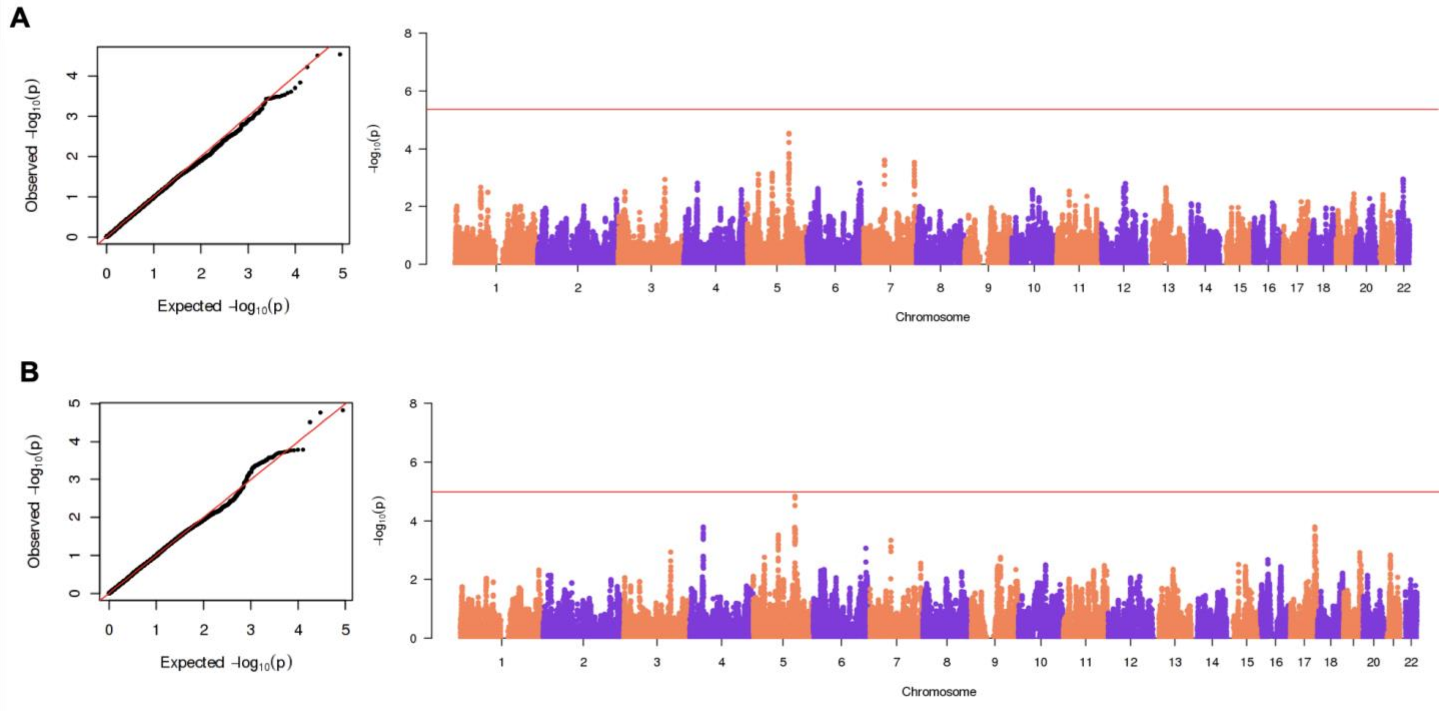

A) Admixture mapping using all cases and all HHCs (N = 2105 cases with active TB and 1320 HHCs with latent TB). We tested the association of local native Peruvian ancestry with TB progression risk after correction for age, sex, global West African, East Asian, and European ancestry proportions, and genetic relatedness (Right)**.** Each dot represents a local ancestry interval including 20 SNPs (N = 44470 intervals). No loci passed the genome-wide significance threshold. Redline: genome-wide significance threshold set using 1000 permutations (4.3×10^{-6}). We observed suggestive evidence of association at 5p23.2 (OR= 1.34 (1.17-1.53), p-value=2.9×10^{-5}. We observed no inflation in the association p-values (left, quantile-quantile plot of admixture mapping p-values on the left, lambda = 0.94). **B)** Similar to A for a subset of cases and controls. To avoid noise introduced by potential phenotypic heterogeneity, we restricted our cohort to cases with microbiologically confirmed TB (N=2043) and HHCs who were TST positive at baseline and did not progress to active TB over the one year follow up (N=950). The locus on

5p23.2 got stronger and closer to the genome-wide significance threshold (OR= 1.39 (1.20-1.61), p-value=1.5×10^{-5}). Redline: genome-wide significance threshold set using 1000 permutations (1.0×10^{-5}). We observed no inflation in the association p-values (quantile-quantile plot of admixture mapping p-values on the left, lambda = 0.99).

Supplementary Tables

Table S1: Global ancestry inference results using Global ancestry inference results using different reference panels, Related to Figure 2A.

input	number of variants for analysis	NAT	EUR	AFR	ASI
LIMAA+1KG	120,630	0.81(0.15)	0.15(0.11)	0.03(0.07)	0.01(0.03)
LIMAA+1KG+Reich	22,198	0.80(0.15)	0.16(0.11)	0.03(0.07)	0.01(0.03)

ADMIXTURE (Alexander et al., 2009) (K = 4) analysis was done on data from Peruvians included in this study (LIMAA, N = 3425) merged with the data from populations from the 1000 Genomes Project phase 3 (1KG, N = 2054) and/or the data from Siberian and Native American populations (N = 738) as the reference panel. The values are presented in the following format: average (standard deviation). NAT: native Peruvian ancestry, EUR: European ancestry, AFR: West African ancestry, ASI: East Asian ancestry.

Table S2: Ancestral proportions when using a larger number of ancestral clusters (K = 4-7) in the ADMIXTURE analysis, Related to Figure 2A.

ADMIXTURE	Reference	NAT	EUR	AFR	EAS	SAS	Chibchan Paezan	Uralic Yukaghir
K=4	Reich + 1 KG	0.80 (0.15)	0.16 (0.11)	0.03 (0.07)	0.01 (0.03)			
K=5	Reich + 1 KG	0.80 (0.15)	0.15 (0.1)	0.03 (0.07)	0.01 (0.03)	0.01 (0.01)		
K=6	Reich + 1 KG	0.71 (0.17)	0.15 (0.1)	0.03 (0.07)	0.01 (0.03)	0.01 (0.01)	0.08 (0.09)	
K=7	Reich + 1 KG	0.68 (0.19)	0.14 (0.01)	0.03 (0.07)	0.01 (0.03)	0.01 (0.01)	0.12 (0.12)	0.01 (0.01)

ADMIXTURE (Alexander et al., 2009) analysis was done on data from Peruvians included in this study (LIMAA, N = 3425) merged with the data from populations from the 1000 Genomes Project phase 3 (1KG, N = 2054) and the data from Siberian and Native American populations (N = 738). The merged dataset includes 22198 variants. Increasing the number of clusters in the ADMIXTURE analysis revealed the finer substructure within each of the four main ancestral populations. NAT: native Peruvian ancestry, EUR: European ancestry, AFR: West African ancestry, ASI: East Asian ancestry. Population labels were assigned based on comparison with population labels in the reference populations.

Table S3: Self-reported race and ethnicity in our cohort among individuals with > 0.9 estimated native Peruvian genetic ancestry (N = 985), Related to Figure 3.

**Self-reported race**	**Count**
American indian + White	762
American Indian	175
White	39
Black	1
American indian + Black	1
Asian	3
Black + White	1
American indian + White + Asian	0
NA	3
**Self-reported ethnicity**	**Count**
Latino	979
Not Latino	4
NA	2

Table S4: Self-reported race and ethnicity in our cohort among individuals with < 0.5 estimated native Peruvian genetic ancestry (N = 140), Related to Figure 3.

Self-reported race	Count
American indian + White	80
American Indian	10
White	29
Black	16
American indian + Black	3
Asian	1
Black + White	0
American indian + White + Asian	0
NA	1
**Self-reported ethnicity**	**Count**
Latino	138
Not Latino	1
NA	1

Table S5: Self-reported race in native peruvian ancestry tertiles for cases and controls, Related to Figure 3.

NAT tertile	Self-reported race	Total	TB case	control
1	American Indian + White	800	410	390
	American Indian	151	75	76
	White	131	78	61
	Black	36	20	16
	American Indian + Black	5	1	4
	Asian	1	1	0
	Black + White	4	1	3
	American Indian + White + Asian	1	1	0
	NA	5	3	2
2	American Indian + White	865	553	312
	American Indian	156	111	45
	White	99	61	38
	Black	5	3	2
	American Indian + Black	9	6	3
	Asian	2	1	1
	Black + White	1	1	0
	American Indian + White + Asian	0	0	0
	NA	5	3	2
3	American Indian + White	873	596	227
	American Indian	208	137	71
	White	51	35	16
	Black	1	1	0
	American Indian + Black	1	0	1

	**Asian**	3	3	0
	**Black + White**	1	1	0
	**American Indian + White + Asian**	0	0	0
	**NA**	3	3	0

Table S6: Association between self-reported race and TB progression, Related to Figure 3.

Self-reported race	Odds ratio (CI)	P
**American Indian**	0.94 (0.72 - 1.22)	0.63
**American Indian + Asian + White**	1.36 (0.51 - 3.66)	0.54
**American Indian + Black**	0.80 (0.56 - 1.15)	0.22
**American Indian + White**	0.92 (0.71 - 1.21)	0.56
**Asian**	1.15 (0.72 - 1.84)	0.56
**Black**	0.89 (0.65 - 1.20)	0.43
**Black + White**	0.83 (0.52 - 1.32)	0.42
**White**	0.91 (0.7 - 1.20)	0.51

No category of self-reported race was significantly associated with TB progression ($p > 0.5$).

Table S7: Accounting for self-reported race, Related to Table 3.

Model	NAT OR (CI)	NAT P
TB status ~ age + sex + NAT + SES + (1|HH) + GRM	1.25 (1.18-1.33)	1.1×10^{-13}
TB status ~ age + sex + NAT + SES + self-reported race + (1|HH) + GRM	1.26 (1.18-1.33)	2.3×10^{-13}

Accounting for self-reported race does not change the association between native Peruvian genetic ancestry and TB progression risk. SES: socioeconomic status; NAT: native Peruvian genetic ancestry; race: self-reported race.

Table S8: Association of genetic ancestry and TB progression risk among unrelated individuals (N= 1929 TB cases and 1066 HHCs), Related to Table 3.

Model	Genetic ancestry	$OR_{NAT0.1}$ (CI)	P
NAT + age + sex + SES + (1|HH)	NAT	1.24 (1.17-1.31)	$2.2x10^{-12}$
EUR + age + sex + SES + (1|HH)	EUR	0.78 (0.71-0.84)	$2.2x10^{-9}$
AFR + age + sex + SES + (1|HH)	AFR	0.70 (0.60-0.81)	$1.5x10^{-6}$
ASI + age + sex + SES + (1|HH)	ASI	0.77 (0.59-1.00)	0.05
NAT + EUR + age + sex + SES + (1|HH)	NAT	1.25 (1.11-1.40)	$2.2x10^{-4}$
	EUR	1.01 (0.86-1.19)	0.91
NAT + AFR + age + sex + SES + (1|HH)	NAT	1.22 (1.13-1.32)	$6.4x10^{-7}$
	AFR	0.95 (0.80-1.14)	0.59
NAT + ASI + age + sex + SES + (1|HH)	NAT	1.25 (1.17-1.33)	$8.3x10^{-12}$
	ASI	1.09 (0.84-1.41)	0.53

Table S9: Sex-stratified analysis, Related to Table 3.

Model	NAT OR (CI)	NAT p-value
Male only	1.25 (1.15-1.36)	$1.4x10^{-7}$
Female only	1.29 (1.16-1.43)	$1.0x10^{-6}$

Table S10: Testing the association between native Peruvian ancestry and TB progression risk using larger numbers of ancestral clusters (K = 5-7), Related to Table 3.

	**TB status ~ NAT + sex + age + SES + (1|HH) + GRM**					
	**K = 5**		**K = 6**		**K = 7**	
	**$OR_{NAT0.1}$ (CI)**	**P**	**$OR_{NAT0.1}$ (CI)**	**P**	**$OR_{NAT0.1}$ (CI)**	**P**
**All TB cases vs. HHCs**	1.25 (1.18-1.33)	9.8E-14	1.25 (1.19-1.31)	3.3E-18	1.22 (1.17-1.28)	5.2E-8

Increasing the number of clusters in the ADMIXTURE analysis did not substantively change the association between native Peruvian ancestry and TB progression risk. OR: odds ratio.

Table S11: Association of native Peruvian ancestry with TB progression risk after correction for potential confounders, Related to Table 3.

	**TB status ~ NAT + sex + age + SES + (1|HH) + GRM**		**TB status ~ NAT + sex + age + SES + (1|HH) + GRM + 8 additional covariates**	
	**$OR_{NAT0.1}$ (CI)**	**P**	**$OR_{NAT0.1}$ (CI)**	**P**
**All TB cases vs. HHCs**	1.25 (1.18-1.33)	1.1E-13	1.23 (1.11-1.35)	6.5E-05
**Secondary TB cases vs. HHCs**	1.30 (1.12-1.51)	4.4E-03	NT	NT
**Secondary clustered TB cases vs. HHCs**	1.67 (1.13-2.47)	0.01	NT	NT
**Secondary TB cases vs. primary cases**	1.02 (0.86-1.22)	0.28	NT	NY

The table summarizes different logistic regression models used to test the association of NAT with TB progression risk after accounting for age, sex, socioeconomic status (SES), a random effect to account for individual's household (HH), and genetic relatedness (GRM). We performed a series of sensitivity analyses to restrict the cohort to secondary or secondary clustered TB cases and their HHCs or to include additional individual-level covariates in the model including African and Asian genetic ancestry proportion, and know TB clinical risk factors smoking status, drinking status, BMI, previous TB status, education level, and BCG vaccination as covariates. For each

analysis. Effect sizes are given for 10% increase in the respective ancestry ($OR_{NAT0.1}$). For analyses using secondary cases, we did not use the larger model as a large number of covariates combined with the small number of samples prevented the models from converging. NT: not tested.

Table S12: Sensitivity analysis using microbiologically confirmed TB cases and their HHCs that were TST positive at baseline, Related to Table 3.

	**TB status ~ NAT + sex + age + SES + (1|HH) + GRM**	
	**$OR_{NAT0.1}$ (CI)**	**P**
**All microbiologically confirmed TB cases vs. TST positive at baseline HHCs**	1.28 (1.20-1.37)	8.6E-14
**Microbiologically confirmed secondary TB cases vs. TST positive at baseline HHCs**	1.46 (1.23-1.74)	2.1E-05
**Microbiologically confirmed secondary clustered TB cases vs. TST positive at baseline HHCs**	1.87 (1.25-2.79)	2.2E-3

To test if the association of native Peruvian ancestry with TB progression risk are affected by potential phenotypic heterogeneity in our cohort, we repeated our analyses in a subset cohort restricted to microbiologically confirmed TB cases and HHCs who were TST positive at baseline and did not progress to TB during the one year follow up. The model parameters and abbreviations are similar to the ones in Table S3. For analyses using secondary and secondary clustered cases HHCs were restricted to those that are from the same households as cases.

Table S13: Variants at the 5p23.2 locus that were nominally associated with TB progression risk in our previously published GWAS, Related to STAR Methods.

chr	ps	allele1	allele0	af_a1	beta_a1	se	p_score
5	125868684	T	C	0.074	-7.49E-02	2.27E-02	9.94E-04
5	125899889	A	G	0.26	-4.05E-02	1.39E-02	3.80E-03
5	125899854	T	G	0.258	-4.05E-02	1.39E-02	3.81E-03
5	125867838	A	G	0.065	-6.23E-02	2.36E-02	8.40E-03
5	125880589	C	T	0.263	-3.16E-02	1.29E-02	1.43E-02
5	125873409	T	C	0.255	-3.13E-02	1.29E-02	1.54E-02
5	125910033	T	C	0.627	3.17E-02	1.31E-02	1.64E-02
5	125878909	T	A	0.261	-2.93E-02	1.26E-02	2.05E-02
5	125868121	A	C	0.033	-7.90E-02	3.44E-02	2.18E-02
5	125928737	T	C	0.024	-8.50E-02	3.71E-02	2.19E-02
5	125878908	A	C	0.262	-2.85E-02	1.26E-02	2.37E-02
5	125872017	A	G	0.651	2.73E-02	1.21E-02	2.40E-02
5	125921498	A	G	0.029	-7.66E-02	3.42E-02	2.51E-02
5	125872243	A	G	0.329	-2.72E-02	1.22E-02	2.52E-02
5	125872150	C	T	0.654	2.70E-02	1.21E-02	2.58E-02
5	125932722	A	G	0.042	-6.60E-02	2.96E-02	2.60E-02
5	125874804	T	G	0.736	2.83E-02	1.27E-02	2.61E-02
5	125871826	AAG	A	0.646	2.73E-02	1.23E-02	2.66E-02
5	125926839	C	T	0.029	-7.61E-02	3.43E-02	2.67E-02
5	125917249	T	G	0.024	-8.17E-02	3.69E-02	2.69E-02
5	125899475	G	A	0.41	2.66E-02	1.20E-02	2.70E-02
5	125879931	A	G	0.652	2.61E-02	1.19E-02	2.82E-02

5	125879839	T	C	0.652	2.58E-02	1.19E-02	2.99E-02
5	125919066	A	G	0.027	-7.72E-02	3.56E-02	3.02E-02
5	125877659	G	GA	0.052	6.61E-02	3.06E-02	3.05E-02
5	125878954	C	CA	0.343	-2.56E-02	1.19E-02	3.08E-02
5	125915744	C	T	0.027	-7.70E-02	3.57E-02	3.10E-02
5	125915346	TAAA	TA	0.026	-7.73E-02	3.58E-02	3.11E-02
5	125859065	A	G	0.053	5.32E-02	2.46E-02	3.11E-02
5	125915922	G	C	0.024	-7.97E-02	3.70E-02	3.12E-02
5	125915374	T	G	0.026	-7.64E-02	3.57E-02	3.23E-02
5	125908599	G	C	0.027	-7.61E-02	3.57E-02	3.32E-02
5	125908586	G	A	0.027	-7.60E-02	3.57E-02	3.33E-02
5	125882825	T	C	0.229	-3.28E-02	1.54E-02	3.38E-02
5	125883472	G	A	0.747	3.11E-02	1.45E-02	3.40E-02
5	125917657	A	G	0.024	-7.83E-02	3.70E-02	3.43E-02
5	125879850	C	G	0.344	-2.50E-02	1.19E-02	3.52E-02
5	125914326	A	G	0.026	-7.45E-02	3.57E-02	3.67E-02
5	125886071	T	C	0.049	-5.58E-02	2.67E-02	3.70E-02
5	125934624	C	G	0.018	-9.32E-02	4.49E-02	3.87E-02
5	125932715	AT	A	0.018	-9.32E-02	4.49E-02	3.88E-02
5	125959700	GT	G	0.381	2.58E-02	1.24E-02	3.89E-02
5	125916297	AT	A	0.03	-6.97E-02	3.38E-02	3.93E-02
5	125931041	A	G	0.018	-9.31E-02	4.50E-02	3.94E-02
5	125879549	T	C	0.334	-2.45E-02	1.19E-02	3.98E-02
5	125874386	TACTA	T	0.336	-2.45E-02	1.19E-02	4.00E-02
5	125874993	G	A	0.336	-2.44E-02	1.19E-02	4.04E-02

5	125874449	A	G	0.336	-2.44E-02	1.19E-02	4.10E-02
5	125883228	C	A	0.2	-3.36E-02	1.64E-02	4.15E-02
5	125916032	A	G	0.029	-6.88E-02	3.39E-02	4.21E-02
5	125881250	T	C	0.033	-6.85E-02	3.37E-02	4.21E-02
5	125874334	C	T	0.336	-2.42E-02	1.19E-02	4.26E-02
5	125874286	G	A	0.013	-9.96E-02	4.91E-02	4.27E-02
5	125877635	G	A	0.334	-2.42E-02	1.19E-02	4.28E-02
5	125931797	CT	C	0.019	-9.06E-02	4.46E-02	4.28E-02
5	125867809	T	C	0.093	-4.20E-02	2.08E-02	4.34E-02
5	125915779	A	G	0.029	-6.84E-02	3.39E-02	4.36E-02
5	125873989	T	C	0.255	-2.58E-02	1.28E-02	4.39E-02
5	125915685	T	TTTAATTG	0.029	-6.82E-02	3.39E-02	4.40E-02
5	125900972	T	C	0.215	-3.02E-02	1.49E-02	4.45E-02
5	125913012	G	GT	0.024	-7.44E-02	3.70E-02	4.46E-02
5	125912120	G	A	0.024	-7.39E-02	3.70E-02	4.61E-02
5	125918148	G	A	0.034	-6.24E-02	3.13E-02	4.65E-02
5	125890991	T	G	0.047	-5.46E-02	2.74E-02	4.71E-02
5	125898345	G	T	0.216	-2.87E-02	1.45E-02	4.79E-02
5	125894437	A	C	0.047	-5.44E-02	2.74E-02	4.81E-02
5	125892829	T	C	0.047	-5.42E-02	2.74E-02	4.87E-02
5	125921381	A	G	0.03	-6.62E-02	3.37E-02	4.96E-02
5	125887496	G	A	0.117	-3.49E-02	1.76E-02	4.96E-02

We observed suggestive evidence of association between local propositions of native Peruvian ancestry and TB progression risk at 5p23.2 in a region that encompasses 100Mb. This region includes 69 variants that were nominally associated with TB progression risk in our cohort in a

previously published GWAS that was performed in the same cohort as the one we used in this study.
